# Influence of Lipoxin-A4 Treatment on Cytokine, Chemokine Genes Expression, and Phenotypic Distribution of Lymphocyte Subsets During Experimental Liver Fibrosis

**DOI:** 10.5152/eurasianjmed.2022.20030

**Published:** 2022-02-01

**Authors:** Zeynal Mete Karaca, Elçin Latife Kurtoğlu, Mehmet Gül, Başak Kayhan

**Affiliations:** 1 Department of Medical Biology and Genetics, İnönü University Faculty of Medicine, Malatya, Turkey; 2 Department of Histology and Embryology İnönü University Faculty of Medicine, Malatya, Turkey; 3 Transplantation Immunology Laboratory, Department of General Surgery, Liver Transplantation Institute, İnönü University, Malatya, Turkey; 4 Department of Pharmaceutical Microbiology, Anadolu University Faculty of Pharmacy, Eskişehir, Turkey

**Keywords:** Immune response, lipoxin-A4, liver fibrosis

## Abstract

**Objective:** Lipoxins are anti-inflammatory, pro-resolving molecules that are secreted by immune cells such as neutrophils and macrophages. Lipoxins are a metabolite of the arachidonic acid pathway that resolve inflammation in fibrotic liver by producing several anti-inflammatory molecules. In this study, phenotypic distribution activation markers of lymphocytes in the spleen and expression levels of chemokines (chemokine (C-X-C motif) receptor 3, chemokine (C-X-C motif) ligand 10) cytokines (interferon gamma, tumor necrosis factor alpha, interleukin-6, interleukin-10) in the liver of lipoxin A4-treated fibrotic mice were investigated.

**Materials and methods:** Liver fibrosis was induced in BALB/c mice by thioacetamide administration. Lipoxin A4 was administered during last 2 weeks of induction. Fibrosis level was determined by using Knodell scoring. Lymphocytes were identified by flow-cytometry. Expression levels of genes were measured by quantitative real time polymerase chain reaction in liver homogenates.

**Results:** Lipoxin A4 treatment caused an elevation of T-lymphocyte percentage in the spleen. Interestingly, administration of lipoxin A4 significantly reduced B-lymphocyte population in spleen of fibrotic group. CD8^+^ cytotoxic T cell frequency significantly reduced in thioacetamide-induced mice; however, lipoxin A4 administration increased that percentage significantly. Lipoxin A4 treatment significantly reduced frequency of activated (CD8^+^CD69^+^) cytotoxic T cells. Expression levels of chemokines significantly reduced in the liver after lipoxin A4 treatment. While expression levels of interferon gamma, tumor necrosis factor alpha, and interleukin-6 significantly reduced in the liver after lipoxin A4 treatment, an anti-inflammatory cytokine interleukin-10 expression was almost at similar levels in all experimental groups.

**Conclusion:** Lipoxin A4 performs its anti-inflammatory effect by reducing the frequency of activated T cells and expression levels of chemokines cytokines responsible from inflammatory immune response in the liver.

## Main Points

- Lipoxin A4 (LXA4) reduces inflammation in the liver and preserves the regeneration capacity of liver.
- Systemic administration of LXA4 shows that anti-inflammatory effect by reducing the frequency of activated cytotoxic T cells in systemic circulation.
  Additionally, LXA4 administration reduced the expression of CXCR3 chemokine receptor in which is mainly expressed on activated cytotoxic T cells in fibrotic liver tissue.
  Since CXCR3 is activated by CXCL10 (IP-10), we observed that LXA4 administration decreased the expression of CXCL10 in fibrotic liver tissue, and accordingly, there was a decrease in the expressions of inflammatory cytokines IFN-g, TNF-a and IL-6.Inflammatory cytokines TNF-a, IFN-g and IL-6 expressions reduced significantly after LXA4 administration.
- Systemic administration of LXA4 reduces the inflammation by reducing the frequency and activation genes expression of T cells in the liver during an experimental fibrosis.

## Introduction

Liver fibrosis is the excessive accumulation of extracellular matrix (ECM) proteins, including collagen that occurs in most types of chronic liver diseases. Advanced liver fibrosis results in cirrhosis, liver failure, and portal hypertension and often requires liver transplantation.^1-3^ There is no standard treatment for liver fibrosis. Although experimental studies have revealed targets to prevent fibrosis progression in rodents, the efficacy of most treatments has not been proven in humans.^4-6^ The ideal anti-fibrotic therapy would be the one which is liver-specific, well tolerated when administered for prolonged periods of time, and effective in attenuating excessive collagen deposition without disturbing normal ECM synthesis.^6^

The lipoxins are synthesized from arachidonic acid metabolism by the action of the lipoxygenase enzymes.^7^ Lipoxin A4 (LXA4) was first identified by Serhan et al^8^ as an interaction product of activated leukocytes in 1984. Endogenic LXA4 biosynthesis occurs via interaction of leukocytes with epithelium, endothelium, or platelets. Lipoxin A4 acts as an anti-inflammatory mediator by inhibiting neutrophil infiltration and transmigration at sites of inflammation and suppressing leukocyte–endothelial interactions.^9^ Lipoxin A4 shows its anti-inflammatory effect both by reducing inflammatory cytokine levels and reducing chemokine secretion in tissue-specific manner. In case of inflammatory response, Sodin-Semri et al^10^ showed that LXA4 in activated synovial fibroblasts inhibits the synthesis of inflammatory cytokines IL-6, matrix metalloproteinase-3, and a chemokine IL-8, in vitro. For the latter one, Gewirtz et al^11^ demonstrated that pathogen-induced chemokine secretion was inhibited by LXA4 analogs. In order to prove that they infected monolayers of T84 intestinal epithelial cells with *Salmonella typhimurium* and detected an elevation on secretion of pathogen-elicited epithelial chemoattractant and IL-8 chemokines. Stable LXA4 analogs administration inhibited *S. typhimurium*-induced secretion of both IL-8 and pathogen-elicited epithelial chemo attractants.

Recently, it has been invented that parenteral administration of LXA4 reduces inflammation in the liver and that also preserves the regeneration capacity of liver.^12,13^ However, the role of cellular immune response, especially lymphocytes in systemic circulation, and the role of chemokines and cytokines at molecular level in the liver are not known. In this study, the phenotypic distribution together with activation status of lymphocytes and gene expression levels of chemokines [chemokine (C-X-C motif) receptor 3 (CXCR3), chemokine (C-X-C motif) ligand 10 (CXCL10)], and cytokines [interferon gamma (IFN-γ), tumor necrosis factor alpha (TNF-α), and interleukin (IL)-6 and IL-10)] in the liver during fibrosis and after LXA4 treatment were investigated.

## Materials and Methods

### Animals

For experimental animal studies, male, BALB/c mice at 5-6 weeks of age were used. Experimental animal studies started with the permission of İnönü University Experimental Animal Production and Research Center (Ethics Committee Permission No: 2014/A-15) and the studies were carried out in accordance with ethical rules of practices of İnönü University Experimental Animal Production and Research Center.

### Experimental Liver Fibrosis

Thioacetamide (TAA) (J&K Chemicals, Pforzheim, Germany) was dissolved in phosphate buffer solution (150 mM NaCl, 30 mM KCl, 15 mM Na_2_HPO_4_, 2 mM KH_2_PO_4_, pH 7.4) (PBS [Sigma Chem. Co., Mo; USA]) before injecting to animals. Thioacetamide at a concentration of 100 mg/kg was administered intraperitoneally 3 days a week for 12 weeks. Experimental groups and the number of experimental animals in each group were determined as follows. Healthy animal group consisted of ten animals (represented as naïve); TAA administered liver fibrosis group consisted of ten animals (represented as TAA); only LXA4-given group composed of ten animals (represented as LXA4); TAA and LXA4 given group consisted of ten animals (indicated as TAA + LXA4).

### Preparation and Use of Lipoxin A4

Lipoxin A4 (Cayman Chemical, Mich; USA) was prepared at 5 μg/kg concentration by diluting 5(S),6(R),15R)-trihydroxy-7E,9E,11Z,13E-eicosatetraenoic acid in ice-cold PBS. Lipoxin A4 was administered at 0.5 μg/0.1 mL/mouse every 2 days in a week for 2 weeks, intraperitoneally.^13,14^ Since chronic fibrosis occurs at the end of tenth week, administration of LXA4 was initiated at the last 2 weeks of chronic fibrosis induction. Therefore, during LXA4 injection, TAA injections to experimental mice were continued. The experiment was ended the day after the last injection of LXA4.

### Biochemical Analysis

Alanine aminotransferase (ALT) and aspartate aminotransferase (AST) levels in serum samples were studied in an automated system (Abbot Architect C8000, IL; USA) with quantitative tests. Normal range levels of ALT and AST in m ice were 15-77 U/L and 54-298 U/L, respectively.^15,16^

### Histological Analysis

In order to determine histological damage and its level, liver sections were fixed in 10% neutral buffered formalin and sections of tissues were sliced in 5 μm size by a microtome (RM2145, Leica Microsystems GmbH, Wetzlar, Germany) from paraffin blocks. Following that, samples were stained with hematoxylin and eosin (H&E). The formation of connective and supporting tissues was performed by trichrome staining method.

Sections were examined by a blind researcher on a light microscope (DFC280, Leica Germany) and imaged in an image analysis system (QWin, Leica, Germany). The Knodell scoring system, which is frequently used in liver fibrosis, was followed for numerical analysis of injury, inflammation level, and regeneration.^17^ Accordingly, the main criteria of evaluation were divided into fragmented necrosis, intralobular degeneration, portal inflammation, and fibrosis main titles. For fragmented necrosis in these main titles, rating scale was none (0 points); light (1 point); medium severity (3 points); significant (4 points); medium-severity and bridge necrosis (5 points); significant and bridge necrosis (6 points); multi-lobe necrosis (10 points). For intralobular degeneration, rating scale was none (0 points), mild (1 point), moderate (3 points), and significant (4 points). For portal inflammation, rating scale was none (0 points), mild (1 point), moderate (3 points), and significant (4 points). For fibrosis, rating scale was none (0 points), portal-distributed fibrosis (1 point), bridging fibrosis (3 points), and significant (4 points). Histological points were the sum of the 4 assessments listed above.

### Cell Isolation and Immune Phenotyping

Spleens were collected under aseptic conditions. Single-cell suspension was prepared by mechanical disruption and straining through nylon mesh. Erythrocyte lysis solution was applied and following that mononuclear cells were isolated by using Ficoll–Hypaque solution. Number and viability of isolated cells were examined in 0.4% Trypan blue. Cells were at more than 95% viability in all in vitro experiments. After cell counting, 10^6^ cells/spleen were dispersed in the tubes and stained with fluorescent dye labeled antibodies. Antibodies used in labeling and fluorescent dyes they are marked with are as follows: fluorescein isothiocyanate (FITC)-labeled anti-mouse CD3ε (BD BioScience, San Jose, Calif, USA); phycoerythrin (PE)-labeled anti-mouse CD19 (BD BioScience); PE-labeled anti-mouse CD4 (BD BioScience); biotin-labeled anti-mouse CD127 (BD BioScience); peridinin chlorophyll-labeled anti-mouse CD4 and CD8 (BD BioScience); FITC-labeled anti-mouse CD69 (BD BioScience); FITC-labeled streptavidin (BD BioScience); FITC-labeled anti-mouse CD3 (BD BioScience); anti-mouse Fc block (Clone 93, ebioscience, Thermo Fischer Scientific Inc, Waltham, M, USA). Prior to staining of the cells, 1/100 diluted Fc block was applied to all cells to prevent cross-linking and then the cells were labeled in suitable tubes and matched with appropriate fluorescently labeled antibodies as mentioned previously.^18^ Analysis of cells was performed on a flow cytometer (FACSCanto II, BD BioSciences). Data were analyzed in BD FACS DIVA Software v6.1.3 (BD BioScience) analysis software. In each assay, 20 000 cells were analyzed.

### Gene Expression

Total RNA was extracted from mouse liver tissues using a RNeasy Plus Mini Kit (Qiagen GmbH, Hilden, Germany). RNA presence and quality were determined by using nanodrop device (Maestro NanoDrop; Taiwan). RNA isolations were repeated according to RNA/DNA ratios. In order to obtain cDNA, an RT^2^-First Strand Kit was used according to manufacturer’s instructions (Qiagen). The cDNAs were stored at −20^o^C until used for reverse transcription polymerase chain reaction (RT-PCR). Reverse transcription polymerase chain reaction assay was performed by using an RT^2^SYBR Green qPCR Master mix (Qiagen, Germany) according to manufacturer’s instructions with minor modifications as we mentioned previously.^19^ Briefly, for each gene (IL-6, TNF-α, IFN-γ, IL-10, CXCR3, CXCL10, GAPDH), PCR mix was prepared. Reverse transcription polymerase chain reaction conditions were determined as 95^o^C for 10 minutes (1 cycle), 40 cycles of 15 seconds at 95^o^C and 30 seconds at 60^o^C in RotorGene. Polymerase chain reaction products were visualized on 2% agarose gel electrophoresis. For that purpose, 100bp standard DNA sample and 15 mL of PCR product with 3 mL of 6× loading dye for each gene were mixed and run for 30 minutes at 100 Volts. Gel images were obtained and recorded on Bioimaging systems (DigiDoc-It System, UVP Bioimaging Systems; Calif, USA).

### Statistical Analysis

The data of the research was analyzed using web-based online statistics program.^20^ The suitability of the data for normal distribution was evaluated by Shapiro–Wilk test. Levene test was used to determine whether the variances were homogeneous. One-way Anova test was used for comparisons between groups showing normal distribution. Tukey test was performed in cases where the variances were homogeneous and Tamhane’s T2 test was used in cases where the variances were not homogeneous. For non-normally distributed data, intergroup comparisons were performed using the Kruskal–Wallis H test and then Conover test was used for pair-wise comparisons. The level of significance was set as *P *< .05.

## Results

**Figure 1.**
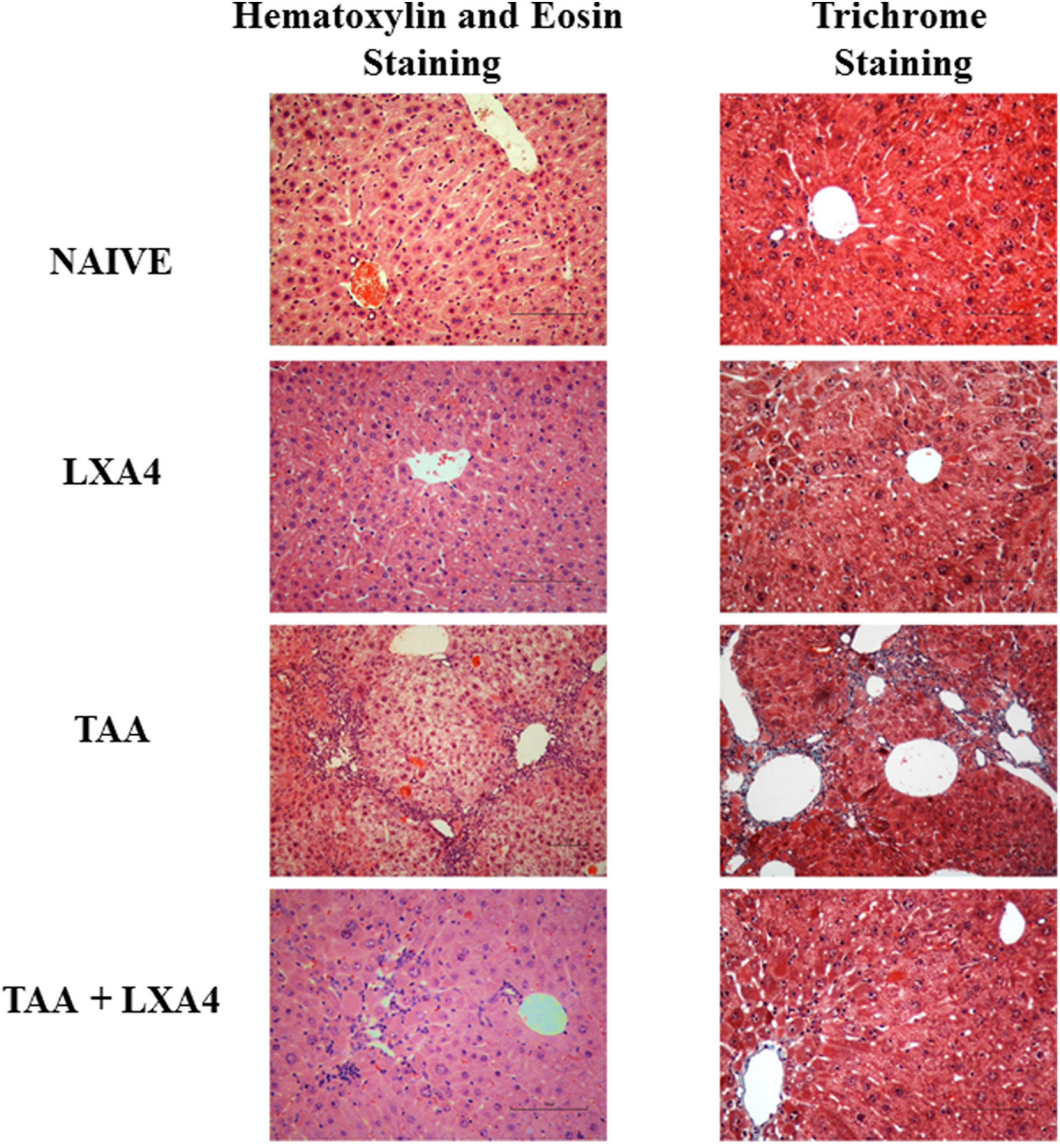
Mononuclear cell infiltration level and the formation of connective and supporting tissues associated with the formation of fibrosis have been determined by H&E and trichrom staining, respectively. Figures show representative histologic views of liver samples for each group. Figures show H&E staining and trichrom staining views with 40× magnifications.

Hematoxylin-eosin staining was used to determine the level of inflammation and fibrosis during histological evaluation of liver tissues. The presence of connective tissue associated with the formation of fibrosis was determined by Gomori’s trichrome staining method (Figure 1).

Histological quantitative evaluation was performed by Knodell scoring scala. Accordingly, liver fibrosis formed by TAA caused significant erosive necrosis, inflammation, lobular necrosis, and fibrosis. Lipoxin A4 administration to fibrotic mice caused a significant reduction on inflammation, erosional necrosis, lobular necrosis, and fibrosis parameters compared to TAA group. In total evaluation, LXA4 injection to TAA-induced fibrotic mice marked significant improvement in liver histology (Table 1, 2).

Alanine aminotransferase and AST are related markers of liver function. Both of them were significantly increased in TAA group. Lipoxin A4 administration to the fibrosis group significantly reduced ALT and AST levels (Table 3).

### Phenotypic Distribution of Mononuclear Cells

**Figure 2.**
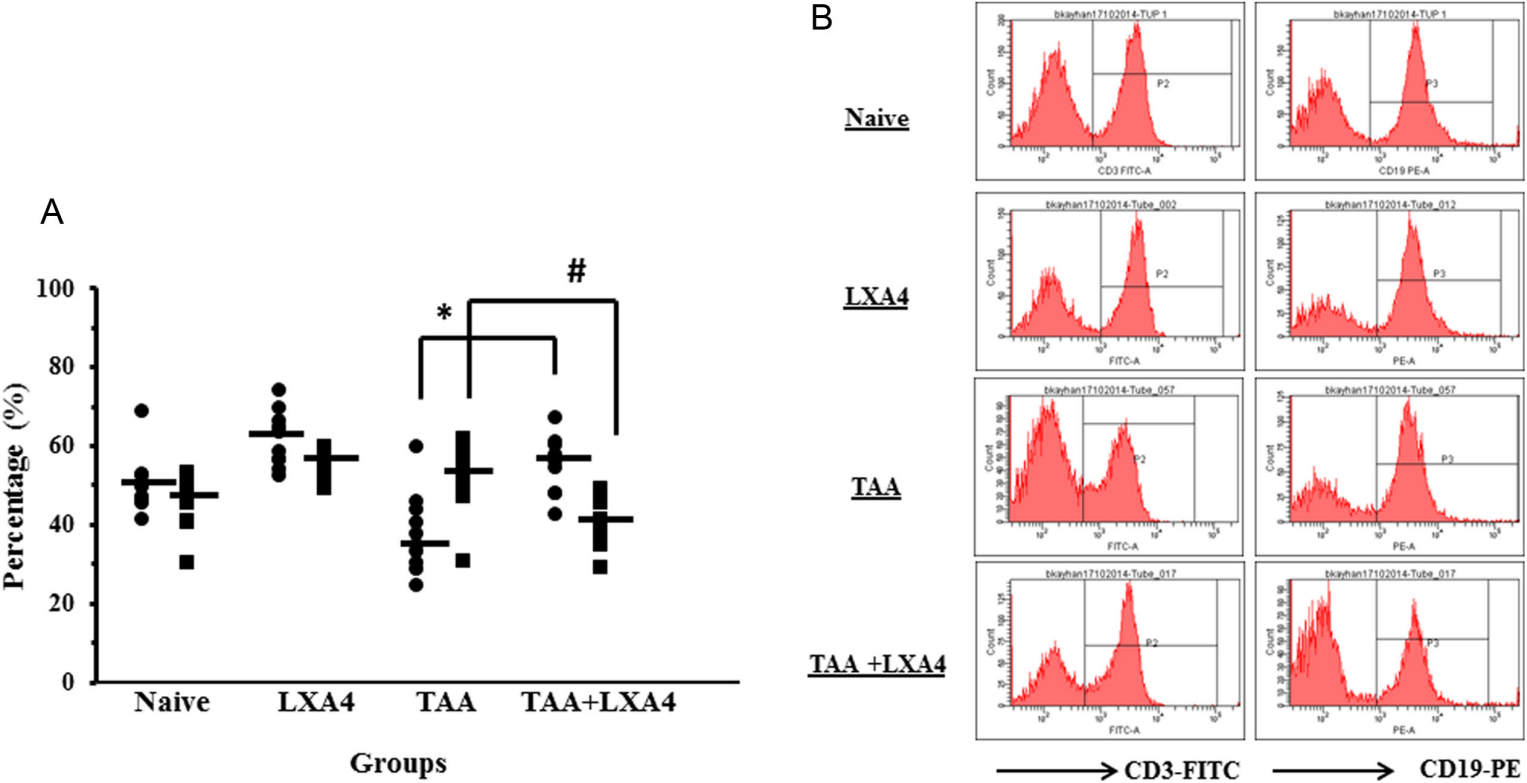
Distribution of T-lymphocyte (CD3^+^) (•) and B-lymphocyte (CD19^+^) (▪) populations in spleen. Figure 2A presents data and mean values for each subject in all experimental groups. Figure 2B, histogram image of representative CD3 and CD19 populations after lymphocyte gate acquisition. The symbols * and # indicate statistically significant parameters of CD3 and CD19, respectively.

The percentage of CD3^+^ T-lymphocyte population significantly reduced during liver fibrosis in comparison to healthy “naïve” group. In contrast, percentage of CD19^+^ B-lymphocytes significantly elevated during liver fibrosis compared to the naive group. While LXA4 injection led to an increase in CD3^+^ T-lymphocyte percentage during fibrosis, interestingly that treatment significantly reduced the percentage of CD19^+^ B-lymphocytes (Figures 2A, 2B).

**Figure 3.**
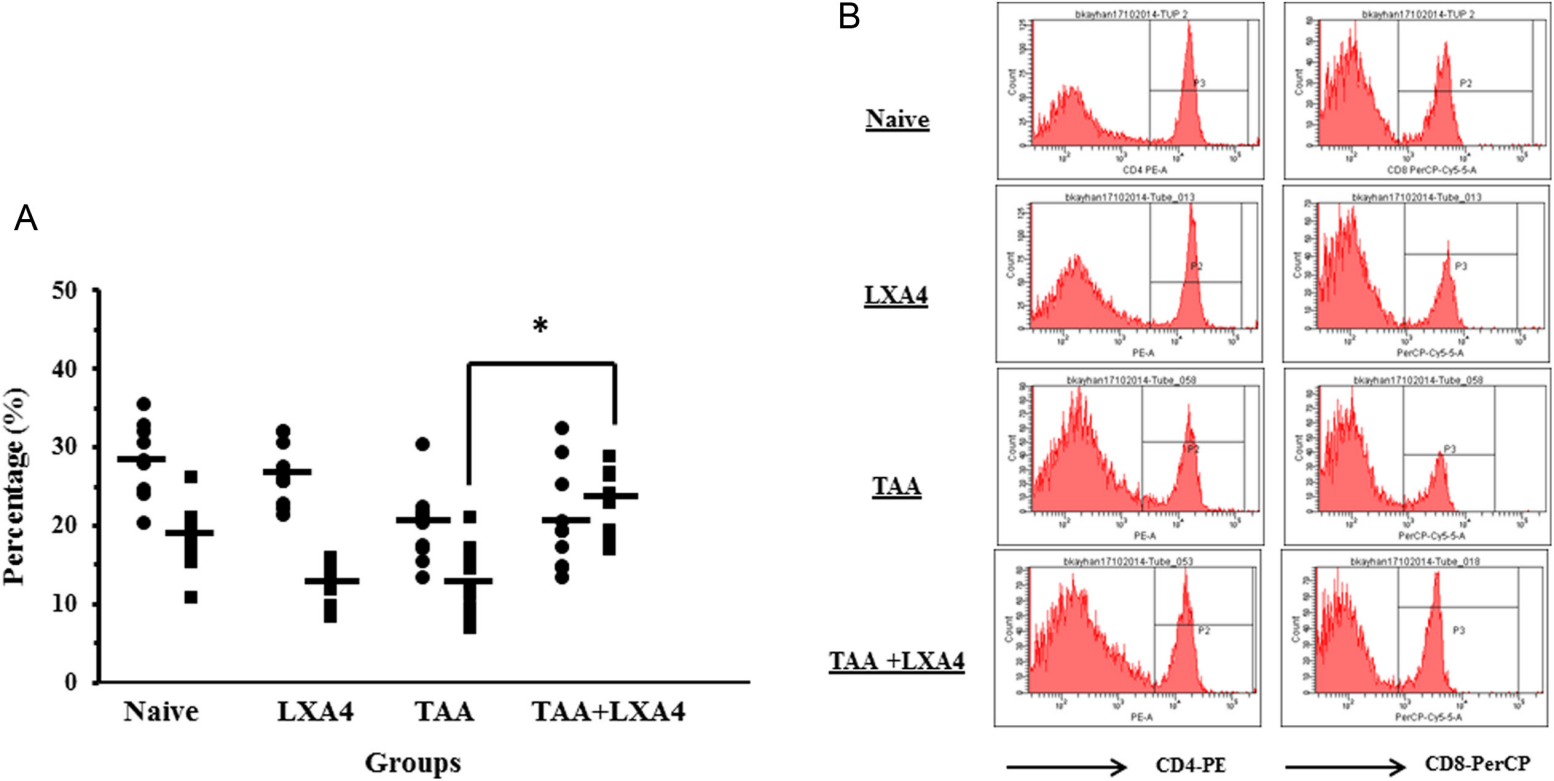
Distribution of helper T-lymphocytes (Th) (CD4^+^) (•) and cytotoxic T-lymphocytes (Tc) (CD8^+^) (▪) cell populations in spleen. Figure 3A presents data and mean values for each subject in all experimental groups. Figure 3B, histogram image of representative CD4 and CD8 populations after CD3 T-lymphocyte gate acquisition. * indicates statistically significant CD8 values between TAA and TAA + LXA4.

Percentage of T-helper cells (CD4^+^CD8^−^) and cytotoxic T cells (CD4^−^CD8^+^) in fibrosis group were significantly lower than the naive group. Treatment with LXA4 significantly increased the cytotoxic T-lymphocyte percentage in comparison to the fibrosis group (Figures 3A, 3B).

**Figure 4.**
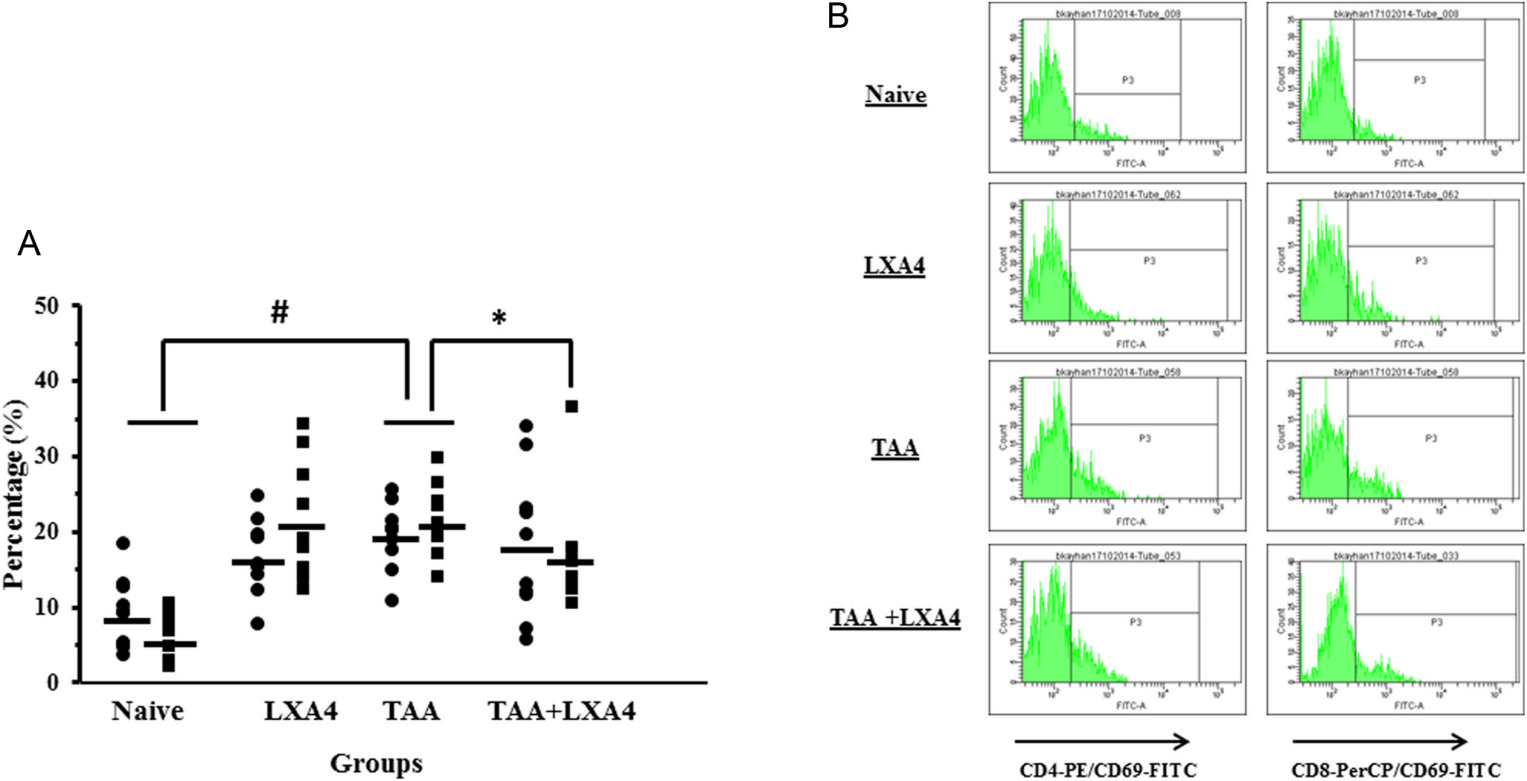
Distribution of CD4^+^CD69^+^ (•) and CD8^+^CD69^+^ (▪) cell populations in spleen. Figure 4A presents data and mean values for each subject in all experimental groups. Figure 4B presents histogram image of representative CD4^+^CD69^+^ and CD8^+^CD69^+^ populations after CD3 T-lymphocyte gate acquisition. * indicates significant difference on CD8^+^CD69^+^ values between TAA and TAA + LXA4 groups. # indicates significant difference on CD4^+^CD69^+^ and CD8^+^CD69^+^ values between Naïve and TAA groups.

In order to to determine the percentage of active lymphocytes percentage of helper and cytotoxic T lymphocytes carrying activation markers, CD69 and CD127 were determined for each group. Liver fibrosis caused significant elevation on the percentage of CD4^+^CD69^+^ and CD8^+^CD69^+^cells in comparison to naïve group in the spleen. After LXA4 treatment, percentage of CD8^+^CD69^+^ cytotoxic T cell population significnatly decreased in comparison to TAA group (Figures 4A, 4B).

**Figure 5.**
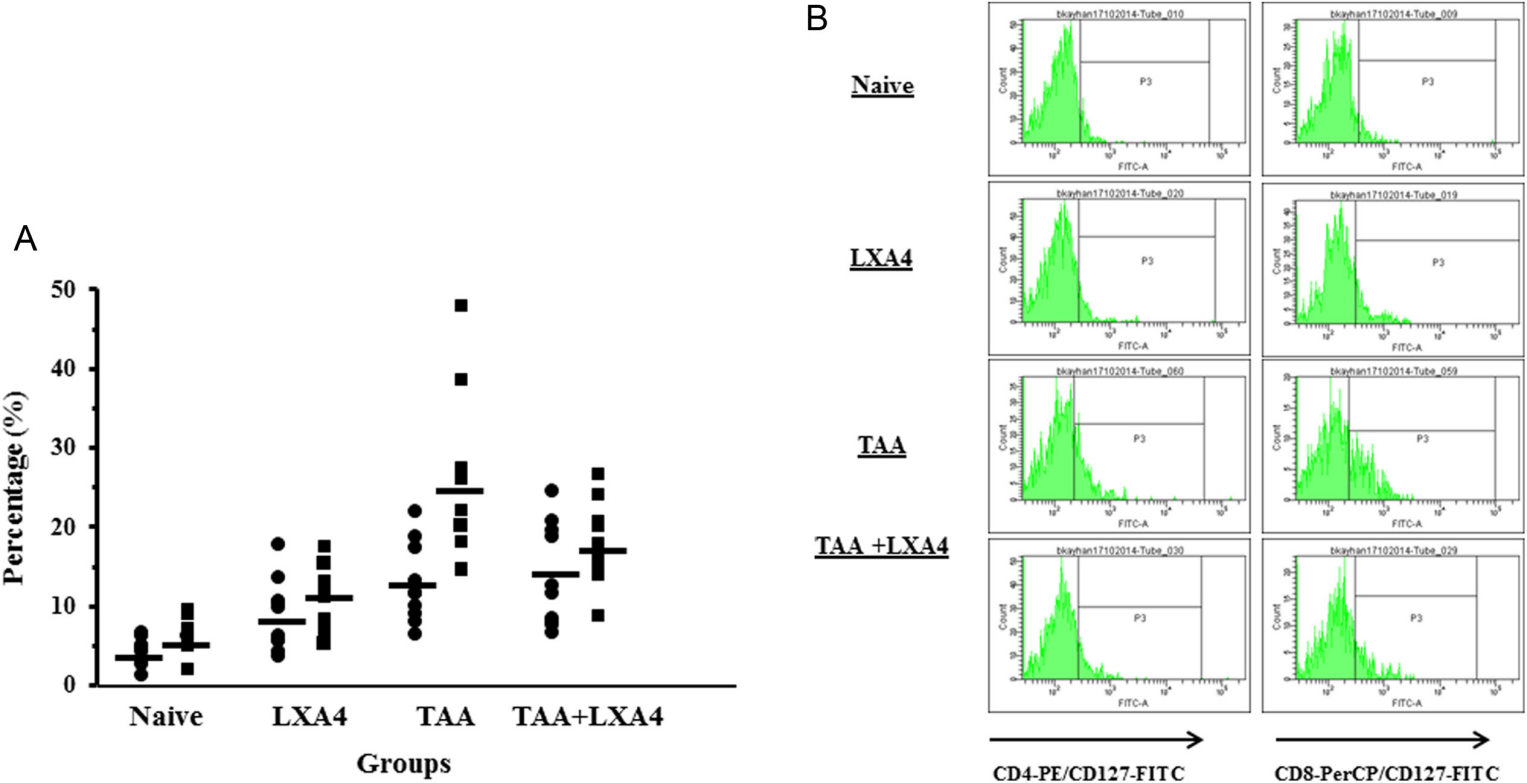
Distribution of CD4^+^CD127^+^ (•) and CD8^+^CD127^+^ (▪) cell populations in spleen. Figure 5A presents data and mean values for each subject in all experimental groups. Figure 4B presents histogram image of representative CD4^+^CD69^+^ and CD8^+^CD69^+^ populations after CD3 T-lymphocyte gate acquisition.

In case of CD127, IL7 receptor alpha chain, liver fibrosis caused an elevation on the percentage of CD4^+^CD127^+^ and CD8^+^CD127^+^ T cells compared to the healthy group. However, we did not observe a significant reduction on those levels after LXA4 treatment (Figures 5A, 5B).

### Chemokine and Cytokine Genes Expression

**Figure 6.**
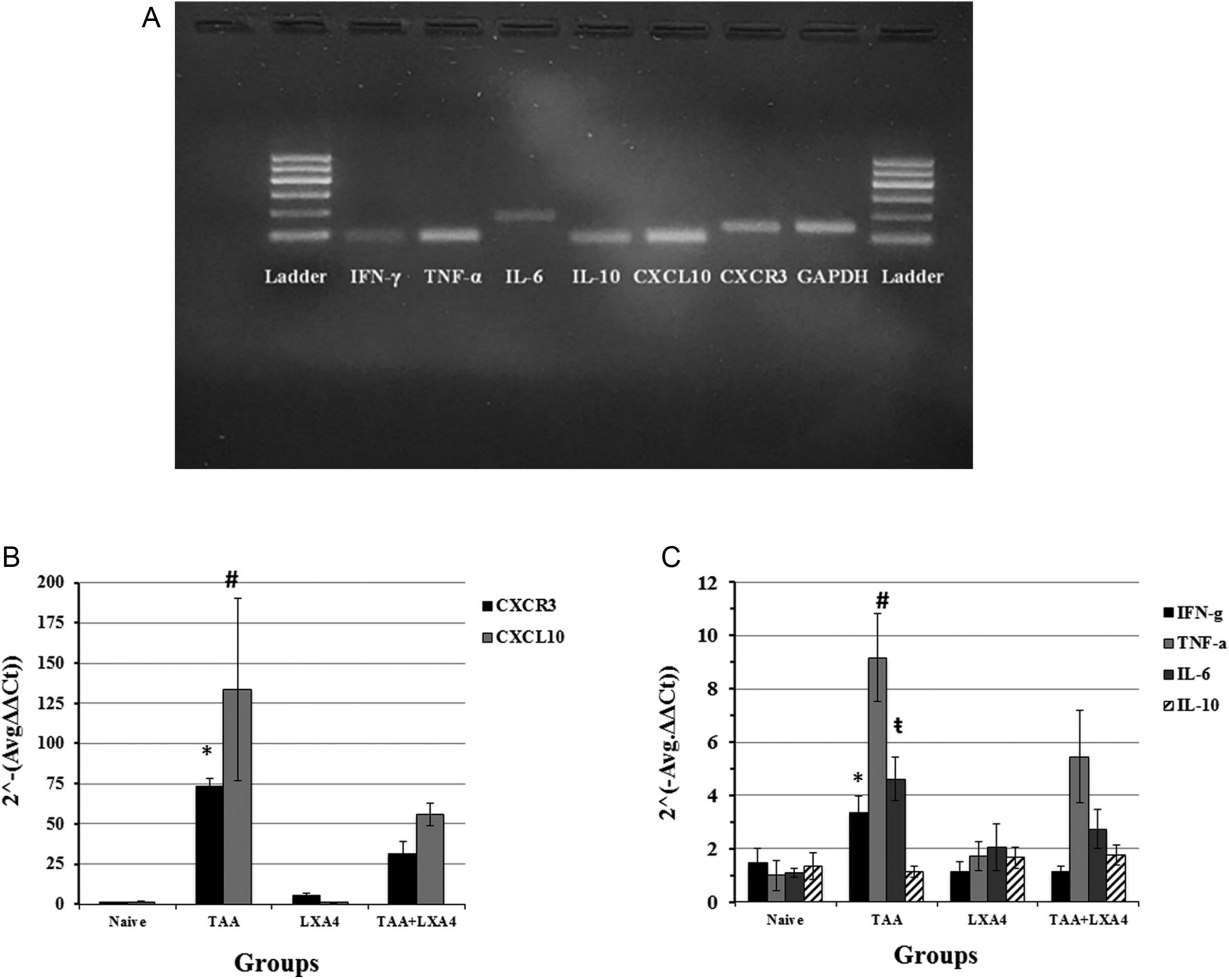
DNA samples after qRT-PCR and visualization of those PCR products at gel electrophoresis (at 2% gel electrophoresis, 45 minutes, 90 V running conditions (A). First and ninth lines 100bp DNA marker (GelPilot, QiaGen); second line is for IFN-γ, third line is for TNF-α; fourth line is for IL-6; fifth line is for IL-10; sixth line is for CXCL10; seventh line is for CXCR3, and eighth line is for GAPDH. qRT-PCR gene expression levels of chemokine (B) and cytokine (C) genes in liver tissues. The data represent the mean ± SD of 2^(Avg − Δ(ΔCt))^values in 2 separate experiments. In figure 6A, * and # indicate significant differences of CXCR3 and CXCL10 between TAA and TAA+LXA4 groups, respectively. In figure 6B, *, #, and ŧ indicate significant differences of IFN-γ , TNF-α, and IL-6 between TAA and TAA + LXA4 groups, respectively.

Properties of primers used in RT-PCR and size of final products were presented in Table 4. Expression levels of CXCR3 and associated chemokine CXCL10 were determined by qRT-PCR method. After qRT-PCR, DNA samples were visualized at gel electrophoresis (Figure 6A). Expression of CXCL10 and its ligand CXCR3 were significantly increased in fibrotic liver. While treatment with LXA4 reduced CXCL10 expression 2.39 times in comparison to fibrosis group, CXCR3 expression decreased 2.35 times. Interestingly, reduction on CXCR3 and CXCL10 levels after LXA4 treatment did not reach to naïve group levels (Figure 6B).

Expression of inflammatory cytokines (IL-6, TNF-α, IFN-γ) and anti-inflammatory cytokine, IL-10, was investigated in liver homogenates. Lipoxin A4 administration to naïve mice did not induce significant elevation on inflammatory and anti-inflammatory cytokines expression. Thioacetamide-induced fibrosis caused elevation on IFN-γ, IL-6, and TNF-α expression in the liver. Treatment with LXA4 reduced IFN-γ expression level till naïve group’s level. After treatment with LXA4 IL-6 and TNF-α, expression level significantly reduced in comparison to TAA group; however, those reduction did not reach to naïve group’s level (Figure 6C).

## Discussion

Lymphocytes are the main cellular part of adaptive immune response in mammalian immune system. Functional properties of those cells determine the type of immune response during acute and chronic inflammation. Cytokines and chemokines are the key regulators during that process. In order to understand the snapshot view of lymphocytes function in an immune system, investigating the frequencies of those cells expressing activation markers would be significant.

Lipoxins are lipid mediators locally produced via cell-to-cell interactions between leukocytes and resident cells during inflammation to prevent abnormal tissue damage induced by inflammatory response and to limit that damage. Lipoxin A4 exerts its biological actions via G-protein-coupled receptors, ALX/FPR2, expressed on leukocytes and intestinal epithelial cells. Hence, leukocytes expressing these receptors are major targets of LXA4 and inhibits polymorphonuclear cells chemotaxis together with vascular adhesion. On the other hand, they stimulate monocyte chemotaxis and reduce the release of inflammatory cytokines.^21^

It is known that lymphocytes are in close association with fibroblasts in inflamed portal tracts and fibrous septa, suggesting the involvement of the adaptive immune response in fibrogenesis. Animal studies also show that depletion of T cells and B-cells protects animals from liver fibrosis. Novobrantseva et al^22^ has determined that mice deficient in both B and T-cells (RAG^−/−^) are resistant to fibrosis, an acute and chronic models of liver injury. It is well known that malfunction at regulation of nuclear factor kappa-B activation leads to sustained inflammation and a tendency towards to Th1 polarized immune response during fibrosis in the liver. However, there are less amount of knowledge about the systemic immune response during fibrosis in liver. Recently, we determined significant elevation of regulatory T-cells and regulatory B-cells in the spleen during liver fibrosis.^23^ Therefore, it is worthy of notice to investigate the immunophenotyping profile of lymphocytes in systemic immune system. In that study, the frequency of lymphocytes and lymphocyte subgroups in spleen and how much of these cells were activated have been analyzed. Fibrosis-induced inflammation after LXA4 administration did not dissolve completely, that was also observed in phenotypic analysis of lymphoid cells. T-lymphocytes levels were significantly decreased during fibrosis in comparison to healthy controls, whereas treatment with LXA4 increased the percentage of T-lymphocyte to healthy controls level. While an elevation on B-lymphocyte frequency was detected during fibrosis, treatment with LXA4 pulled down that to healthy controls level. Novobreantseva et al^22^ reported that B-lymphocytes have been shown to play an active role in the development of liver fibrosis. They stated that intrahepatic B-cells are phenotypically similar to splenic B2 cells. They express lower levels of CD23 and CD21 and higher levels of CD5. Additionally, they established that B-cells have an impact on fibrosis in an antibody and T-cell-independent manner. The decrease in B-lymphocyte level after LXA4 treatment was found for the first time in our study. However, it is a question of whether this decrease occurs also in the liver or not. Another result of the immunophenotyping study was found in activated T-lymphocyte levels. CD4^+^ and CD8^+^ T-lymphocytes activation during fibrosis increased in comparison to healthy controls. The percentage of activated CD8^+^ T-lymphocytes decreased after LXA4 treatment compared to fibrosis group; however, it is remarkable that it was higher than healthy group. This is probably due to the low level of inflammation in the liver after LXA4 treatment, as determined by histological analysis.

During chronic hepatocyte injury T cells, macrophages and dendritic cells are accumulated by the inflammatory response. Immigration of these cells is regulated by the cytokines and chemokines pattern secreted by activated cells located in the liver.^24-26^ In general, chemokines increase the migration of fibrogenic cells to the site of injury, thereby increasing fibrogenesis through increased cell number and increased inflammation. Accordingly, we observed an elevation on CXCR3 and CXCL10 expression levels during TAA-induced chronic fibrosis. CXCL10 [also known as interferon-gamma stimulating protein-10 (IL-10)] is a chemokine that is effective in aggregating and locating inflammatory cells in the site of tissue damage or infection.^27^ CXCR3, a receptor for CXCL10, is expressed on CD4^+^ and CD8^+^ T-lymphocytes, B-lymphocytes, natural killer, and dendritic cells. Increased CXCL10 and CXCR3 gene expression in chronic hepatitis B and hepatitis C patients were reported to correlate with the severity of the disease. CXCL10 is secreted by hepatocytes in areas with lobular inflammation, which are in contact with CXCR3^+^CD8^+^ T-lymphocytes.^28,29^ Thus, showing lobular degeneration ratio in liver would be crucial to determine the level of fibrosis and its correlation with CXCR3 expression. Although pathologic parameters significantly decreased after LXA4 treatment, those inflammation signs never come to the level of healthy subjects. This may be the reason why reduction on CXCR3 and CXCL10 expression levels after LXA4 treatment was still significantly higher in comparison to healthy group.

Recently, a group of scientists showed that LXA4 treatment during fibrosis significantly reduced TNF-α and IL-17 in liver homogenates. Interestingly, that reduction was not related with elevation on IL-10 in liver homogenates.^12^ In molecular level, LXA4 treatment reduced the expression of TNF-α, IFN-γ and IL-6 significantly. Furthermore, those reduction were not related with a T-cell response switch in the liver since IL-10 expression did not change both in fibrosis and LXA4 treatment groups. Hence, that proves LXA4 performs its anti-inflammatory effect independently of switching T-lymphocyte response. At that point, Machado et al^30^ showed that leukocyte infiltration into liver and brain including expansion of memory IFN-γ-producing T-cells is controlled by a lipoxin regulatory pathway dependent on suppressor of cytokine signaling 2 (SOCS-2) in vivo. According to their hypothesis, lipoxin-dependent induction of SOCS-2 represents a general anti-inflammatory pathway responsible for controlling several innate responses. They observed that LXA4, but not IL-10, failed to inhibit production of IL-12 in dendritic cells isolated from SOCS-2 which indicates distinct regulatory mechanisms triggered by LXA4 and IL-10 related to dependence on SOCS-2.^30^

For the first time in this study, the distribution of cells responsible for cellular immune response and their activation levels during experimental liver fibrosis and its treatment with LXA4 were investigated. However, the decrease in the percentage of activated CD8^+^ lymphocytes and in the expression of inflammatory mediators after treatment with LXA4 only shows that snapshot view. It is not known for how long LXA4 administration maintains this effect. In addition, during the chronic inflammatory response in the liver, the effects of cellular activation in the spleen and the inflammatory response in the spleen on the liver should be investigated.

In conclusion, LXA4 shows anti-inflammatory effect during liver fibrosis by reducing inflammatory cytokine and chemotactic factors related with fibrosis and reducing activated CD8^+^ T cells in systemic circulation.

## Figures and Tables

**Table 1.** Grading Parameters of All Mice in Each Group According to Knodell Scoring Scale

Parameters	Groups			
Naive	LXA4	TAA	TAA + LXA4
**Piecemeal Necrosis**				
None (0)	10 mice	10 mice		
Mild (1)				5 mice
Moderate (3)				4 mice
Marked (4)			1 mouse	1 mouse
Moderate+Bridge Necrosis (5)			3 mice	
Marked+Bridge Necrosis (6)			6 mice	
Multilobular Necrosis (10)				
**Intralobular Degeneration and Focal Necrosis**				
None (0)	9 mice	8 mice		1 mouse
Mild (1)	1 mouse	2 mice		8 mice
Moderate (3)			5 mice	1 mouse
Marked (4)			5 mice	
**Fibrosis**				
No Fibrosis (0)	10 mice	10 mice		2 mice
Fibrous Portal Expansion (1)				6 mice
Bridging Fibrosis (3)			4 mice	2 mice
Cirrhosis (4)			6 mice	
**Portal Inflammation**				
None (0)	9 mice	9 mice		1 mouse
Mild (1)	1 mouse	1 mouse	1 mouse	8 mice
Moderate (3)				1 mouse
Marked (4)			9 mice	

**Table 2.** Quantitative Analysis of Histological Evaluation of Liver Tissue After Knodell Scoring. The Values Indicate the Mean Values of the Analysis Obtained from All Subjects. Total Values Were Evaluated According to Highest Score of Each Parameter. # Symbol Indicates Significant Differences Between TAA and TAA + LXA4

Groups	Pathologic Parameters				Total
Erosion and Necrosis	Lobular Degeneration and Focal Necrosis	Fibrosis	Inflammation
Naive	0.0	0.1	0.0	0.1	0.2/22
LXA4	0.0	0.2	0.0	0.1	0.3/22
TAA	5.5 **#**	3.5 **#**	3.6 **#**	3.7 **#**	16.3/22**#**
TAA+LXA4	2.1	1.1	1.2	1.1	5.5/22

**Table 3.** Serum ALT and AST Levels of Each Group. The Values Indicate the Mean ± Standard Error of the Analysis Results Obtained from All Subjects

Groups	ALT (15-84 U/L^¶^)	AST (54-298 U/L^¶^)
Naive	21.53 ± 5.55	82.2 ± 7.54
LXA4	29.71 ± 8.21	92.48 ± 5.58
TAA	98.12 ± 5.26*	202.5 ± 14.21*
TAA + LXA4	43.19 ± 6.27**#, ** ^ŧ^	114.26 ± 12.28**#,** ^ŧ^

*, **#**, and ŧ symbolize significant differences between Naïve-TAA, TAA-TAA + LXA4 and Naïve-TAA + LXA4 groups, respectively (*P *< .05). ¶ indicates normal range of AST, ALT levels in serum of BALB/c mice (18).

**Table 4.** Properties of Primers Used in qRT-PCR

Gene	Reference Segment Number	Reference Position	Band Range (bp)
*Cxcl10;* Chemokine (C-X-C motif) ligand 10	NM_021274.2	653	89
*Cxcr3;* Chemokine (C-X-C motif) receptor 3	NM_009910.3	1047	132
*Ifng;* Interferon gamma	NM_008337.4	330	95
*Tnf;* Tumor necrosis factor	NM_013693.3	879	93
*Il6;* Interleukin 6	NM_001314054.1	120	178
*Il10;* Interleukin 10	NM_010548.2	104	81
*Gapdh;* Glyceraldehyde 3-phosphate dehydrogenase	NM_008084.3	499	140
